# A Computational Workflow for the Identification of Novel Fragments Acting as Inhibitors of the Activity of Protein Kinase CK1δ

**DOI:** 10.3390/ijms22189741

**Published:** 2021-09-09

**Authors:** Giovanni Bolcato, Eleonora Cescon, Matteo Pavan, Maicol Bissaro, Davide Bassani, Stephanie Federico, Giampiero Spalluto, Mattia Sturlese, Stefano Moro

**Affiliations:** 1 Molecular Modeling Section (MMS), Department of Pharmaceutical and Pharmacological Sciences, University of Padova, Via Marzolo 5, 35131 Padova, Italy; giovanni.bolcato.1@studenti.unipd.it (G.B.); matteo.pavan.7@phd.unipd.it (M.P.); maicol.bissaro@phd.unipd.it (M.B.); davide.bassani.1@studenti.unipd.it (D.B.); mattia.sturlese@unipd.it (M.S.); 2 Department of Chemical and Pharmaceutical Sciences, University of Trieste, Via Licio Giorgeri 1, 34127 Trieste, Italy; eleonora.cescon@phd.units.it (E.C.); sfederico@units.it (S.F.); spalluto@units.it (G.S.)

**Keywords:** fragment-based drug discovery, molecular docking, molecular dynamics, supervised molecular dynamics, protein kinase CK1δ

## Abstract

Fragment-Based Drug Discovery (FBDD) has become, in recent years, a consolidated approach in the drug discovery process, leading to several drug candidates under investigation in clinical trials and some approved drugs. Among these successful applications of the FBDD approach, kinases represent a class of targets where this strategy has demonstrated its real potential with the approved kinase inhibitor Vemurafenib. In the Kinase family, protein kinase CK1 isoform δ (CK1δ) has become a promising target in the treatment of different neurodegenerative diseases such as Alzheimer’s disease, Parkinson’s disease, and amyotrophic lateral sclerosis. In the present work, we set up and applied a computational workflow for the identification of putative fragment binders in large virtual databases. To validate the method, the selected compounds were tested in vitro to assess the CK1δ inhibition.

## 1. Introduction

### 1.1. Protein Kinase CK1δ

Protein kinase CK1δ belongs to the family of CK1 Kinases (Casein Kinase 1), which in turn belongs to the class of Ser-Thr Kinases; seven isoforms of this family were identified in mammals: α, β, γ1, γ2, γ3, δ, ε. All the isoforms of CK1 are constitutionally active and they exhibit activity in monomeric form, They present a highly conserved catalytic domain (unlike in N and terminal C domains), they utilize ATP as a phosphate group donator and they are generally independent of the presence of a cofactor [1].

CK1δ and the other isoforms of the family of CK1 can phosphorylate Ser or Thr residues in sequences such as *(P)Ser/Thr-X_1-2_-Ser/Thr*, where *(P)Ser/Thr* indicates a Ser or Thr pre-phosphorylated residue [2]; CK1, therefore, needs the substrate to bealready phosphorylated. Nevertheless, it has been demonstrated that a set of amino acids with acidic character in the direction of the N-terminal withrespect to Ser/Thr target residue or an acidic residue in position 3 (preferably Asp) can provide for the lack of the pre-phosphorylated amino acid [3,4]. This allows CK1 to act also as a Priming Kinase activating the substrate towards a second enzyme by phosphorylation. Currently, about 140 substrates (in vitro or in vivo) recognized by CK1 have been described [1].

The activity of CK1 isoforms is regulated in different ways. Phosphorylation is the principal strategy adopted for the activity regulation of this family of kinases. CK1δis phosphorylated by kinases such as Akt, PKA, PKCα, CLK2, and Chk1. Moreover, CK1δ can also be subjected to auto-phosphorylation [1,5,6]. Another fundamental aspect in the CK1δ activity regulation is the subcellular compartmentalization, operated through the binding to intracellular structures and other proteins [7,8]. One last mechanism reported in the literature for the CK1δ regulation is the formation of homodimers [9,10].

CK1δ, together with other CK1 isoforms, has been correlated to several neurodegenerative processes [11]; in particular, CK1 seems implied in tauopathies, among which Alzheimer’s disease (AD) is the most representative one.

AD is associated with several cellular processes. The first mechanism described is correlated to Tau protein, which after phosphorylation tends to come off from the microtubules forming aggregates at a cytoplasmatic level, leading to cellular damage. A second mechanism implies instead production and accumulation, with consequent cellular death, of the β-amyloid peptide. This is produced by the cut of its precursor APP (Amyloid Precursor Protein) by β-secretase 1 and γ-secretase enzymes. The implications of CK1 isoforms in pathogenetic processes at the root of Alzheimer’s disease are many. In general, CK1δ proves to be overexpressed in brain tissue, up to 30 times in patients affected by Alzheimer’s disease [12,13].

Concerning Tau protein, initially, it was observed how CK1 turns out to be associated with fibrillar masses of hyperphosphorylated Tau protein (Paired Helical filaments) [14]; in particular, CK1δ seems to be accumulated within these fibrillar masses [15]. Later it was demonstrated how CK1δ can phosphorylate Tau protein causing its separation from microtubules; the residues of Tau phosphorylated by CK1δ are Ser202, Thr205, Ser396, and Thr404 [11,16]. As regards β-amyloid peptide, it was described how this can stimulate the activity of CK1 and CK2 (employing casein as a substrate) [17]. Likewise, there is evidence that CK1 activity would be proportionally correlated to β-amyloid peptide production, since in presence of constitutionally active CK1 forms the amount of thispeptide increases, whereas it decreases in presence of CK1 inhibitors. CK1 interference seems to take place along with the γ-secretase enzyme [18], but it is more likely correlated to CK1ε isoform, than to CK1δ [19].

As regards Parkinson’s Disease, it has been observed how CK1 isoforms phosphorylated Ser129 of α-synuclein [11,20].

Amyotrophic lateral sclerosis (ALS) is another neurodegenerative disease where CK1δ plays a role. Indeed, CK1δ phosphorylated TDP-43 (TransActivate Response DNA Binding Protein 43) at many different residues. TDP-43 is the principal component of the protein aggregates observed in the pathogenesis of ALS [21,22].

### 1.2. Fragment-Based Drug Discovery (FBDD) Principles

FBDD is a strategy used in drug discovery that has gained popularity both in the industrial and academic contexts. In a typical FBDD process a library of polar low molecular weight compounds is screened against a specific target. Usually, the screening is performed by biophysical methods including X-ray crystallography, nuclear magnetic resonance (NMR), thermal shift assay, and surface plasmon resonance (SPR). One of the key factors in the FBDD success is the smaller size of the fragment-like chemical space compared to the size of the drug-like one. The size of the drug-like chemical space has been estimated at around 10^60^ compounds, many orders of magnitude greater than that of the fragment-like compounds’ chemical space [23]. This means that, through the screening of fragments, the portion of chemical space sampled is larger than the one sampled with the screening of drug-like molecules. This will promisingly also allow the attainment ofinnovative scaffolds for drug candidates.

Despite the hit fragments having typically a low affinity, they could be turned into a lead compound that efficiently binds the target. Fragments, having a low molecular weight, establish few interactions with the target; however, the combination of multiple fragments by linking and merging or by decorating them with adequate functional groups (fragment growing) allows thedevelopment of specific and more affine compounds. 

### 1.3. Fragment-Based Drug Discovery and Kinase Inhibitors

Concerning the identification of kinase inhibitors through an FBDD approach, X-ray crystallography has also been largely employed because kinases represent a class of protein that provides good results with this technique. 

The most outstanding example of kinase inhibitors derived from an FBDD approach is Vemurafenib (inhibitor of BRAF) which is an approved drug for the treatment of metastatic melanoma [24]. The discovery of vemurafenib started with an enzymatic assay screening of a fragment library. The hit compounds identified were analyzed through X-ray crystallography, using the structural information so obtained one fragment was chosen for optimization leading at the end to Vemurafenib [25]. Another notable example is Asciminib an allosteric inhibitor of BCR-ABL1 tyrosine kinase, now in phase III clinical trial for resistant chronic myeloid leukemia. This compound was identified from an NMR-based fragment screening; the fragment hits identified were then optimized using In Silico methodologies, X-ray crystallography, and NMR [26,27]. 

Many other Kinase inhibitors derived from FBDD approaches are in clinical trials; for a comprehensive review of FBDD derived drugs that have been approved or which are in clinical trials see [28].

An interesting observation is that the fragments identified often bind at the hinge region of the kinase and maintain this binding mode also in the mature compound. For this reason, the library of compounds tested in the present work has been focalized, using in silico methodologies described in the next sections, to be composed of putative hinge-binding fragments.

### 1.4. Computational Methods in FBDD

Since the dawn of FBDD, computational chemistry has playeda major role in both fragments’ hit identification and in the process of fragment optimization. The MCSS (multiple copy simultaneous search) algorithm [29] was a pioneering method for the study of fragment binding modes in a protein site. Another method for fragment posing based on grand canonical Monte Carlo (GCMC) has been reported [30].

Over the years many in silico methods have been proposed non only for fragment placement prediction but also to aid the fragment optimization process. Software like LUDI [31], HOOK [32], CAVEAT [33], RECORE [34], and many others have been developed for this purpose. Additionally, Schrodinger [35] and CCG [36] implement in their software suites many tools to aid the fragment optimization process. 

Molecular dynamics (MD)-based tools represent the most advanced in silico techniques used in FBDD. The first application of MD to FBDD was the refinement of docking poses, a method note as post-docking [37]. More advanced protocols have also been developed. Nonequilibrium candidate Monte Carlo (NCMC) is an algorithm that has been applied to enhance the sampling of fragment binding modes [38]; this method has been successfully applied to FBDD [39]. Another promising approach is the application of Markov state models to MD simulations, which has proved its potential to FBDD [40]. Recently, Supervised Molecular Dynamics (SuMD) [41] has been applied as a fragment screening tool [42]. 

Molecular docking has also become a routinely used tool in FBDD. While the conformational sampling performs by docking protocols is generally effective in reproducing the correct pose for a ligand, the scoring functions frequently fail in valuating this pose [43], this is especially true for Fragment-like compounds for which many doubts have been raised about the docking applicability [44]. This said, to make the docking results more reliable a consensusdocking approach was used [45], and instead of the scoring function, the poses were evaluated using a pharmacophore model. A post-docking refinement of the poses wasthen performed. A detailed explanation of the computational workflow adopted in the present work is reported in Section 4.1, Section 4.2 and Section 4.3.

## 2. Results

### 2.1. Computational Results

**Figure 1:**
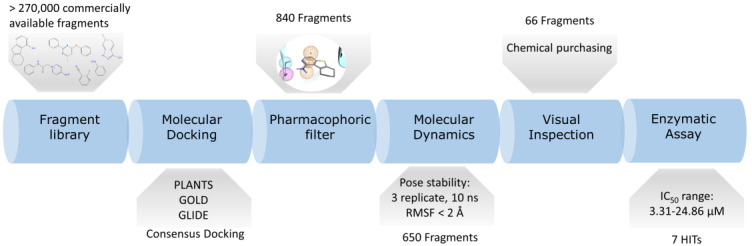
Schematic representation of the workflow adopted in the present work. First the fragments are retrieved from several vendors libraries. After proper preparation, the database is docked using three different docking protocols. the resulting poses have been filtered using a pharmacophore model and only the molecule that fit the model for each protocol have been retained. The poses of these molecules were further refined using MD to assess the stability of the binding mode. the molecules that appear to be stable were finally selected trough visual inspection.

A library of around 272,000 commercially available fragment compounds was screened in silico using an integrated structure-based approach based on different techniques such as molecular docking, molecular dynamics (MD), and pharmacophore filter. The workflow adopted is reported in Figure 1.

At first, three independent docking-based virtual screenings were performed in parallel exploiting three different protocols: PLANTS-ChemPLP, GOLD-ChemScore, and Glide-SP. PLANTS exploits an Ant-Colony Optimization (ACO) algorithm, GOLD a genetic one while Glide performs an exhaustive search. The choice of these three protocols was made to evaluate the virtual library with three orthogonal search algorithms, to minimize the false-positive rate to which traditional docking-based virtual screenings are prone. At the end of each virtual screening, a total of about 13.6 M poses (50 per ligand) was obtained for each protocol. The choice to generate such a great number of poses for each ligand was taken in order not to rely on the scoring function ability to prioritize the best binding mode for each compound, since fragments can have multiple binding modes that are similar from an energetic and qualitative point of view and are therefore difficult to distinguish for scoring functions that are trained upon mature, lead-like, compounds. 

**Figure 2:**
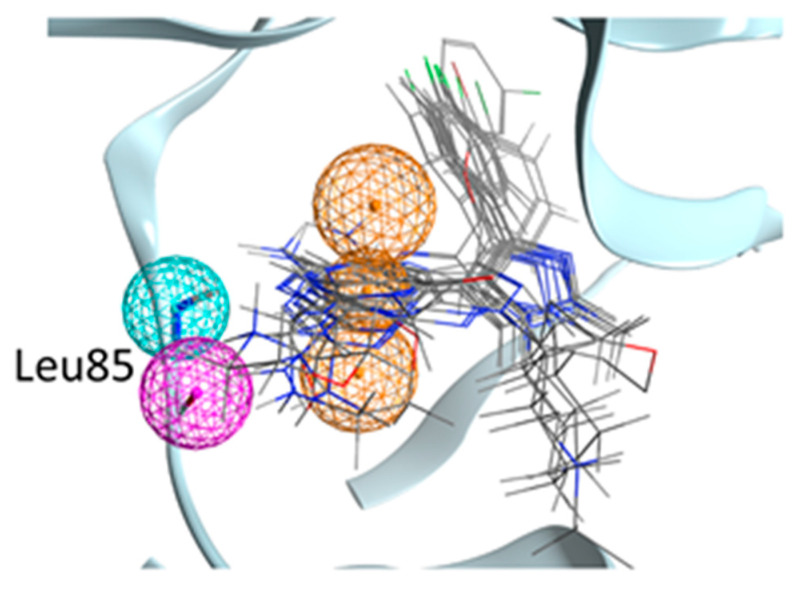
Representation of the pharmacophore model used in the present work. Some representative crystallographic ligands are displayed (not all for clarity). The Pharmacophore model is formed by an aromatic ring (the three orange spheres define the position and its orientation) and two hydrogen bonds with the backbone of Leu85 (an acceptor and one donor).

To filter this huge amount of ligand conformations and retain only the most interesting compounds, we decided to exploit the structural knowledge provided by the 23 Ck1d protein–ligand complexes deposited in the Protein Data Bank and create a pharmacophore filter. This pharmacophore model was built to retain those features which are vital for the interaction with the hinge region of the kinase since these features are the most commonly found across the structures (See Figure 2 for a representation of the Pharmacophore model). The pharmacophore included three features, two of them to guarantee the interaction with Leu85 (a hydrogen bond donor and a hydrogen bond acceptor) and the presence of and a feature for an aromatic ring also in the proximity of the hinge region.

The pharmacophore filter was then applied independently on each pose database generated by the three different docking protocols. Exploiting an approach known as consensus docking, the three libraries containing those ligand conformations that fit the pharmacophore model were merged, retaining only those found within each dataset. After this consensus filtering, only 840 docking poses were left. 

To further filter out those poses characterized by unstable binding modes, a post-docking molecular dynamics refinement was performed (three replicates, 10 ns each). The average Root Mean Squared Fluctuation of atomic positions (RMSF) across the three replicates was used as a cutoff to eliminate those poses characterized by conformational instability over time. After filtering out those ligand conformations with RMSF > 2Å, 650 stable poses were maintained.

With the intent of prioritizing the most interesting compound for in vitro assays, each pose was carefully manually examined. After this visual inspection [46] step, 66 fragments were finally selected to be purchased and tested. The structure of all the 66 fragment compounds tested are reported in supplementary Table S1, while the pose of each of them resulted from the VS pipeline is reported in Video S1. 

### 2.2. Enzymatic Assay Results 

**Figure 3:**
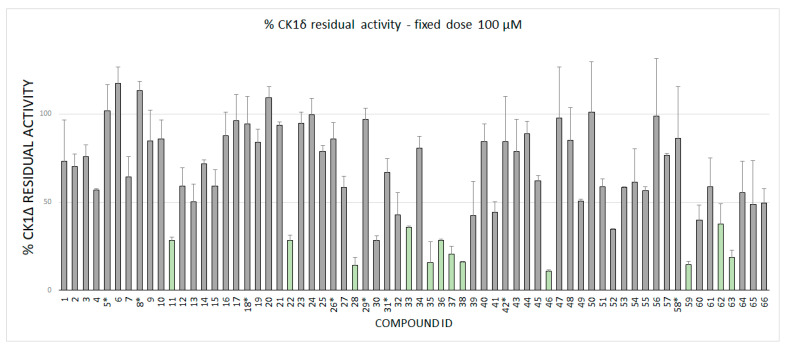
CK1δ residual activity at a concentration of 100 μM of the ligand under examination. The molecules marked with a star (*) has been tested at 50 μM due to solubility issues.

**Figure 4:**
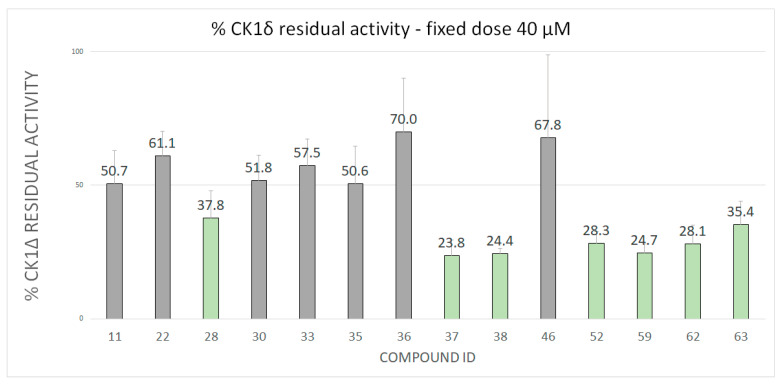
CK1δ Residual activity at a concentration of 40 μM of the ligands that showed a residual activity of less than 40% at 100 μM.

Fragments were tested against CK1δ using a luminescent-based assay. Compounds were evaluated at a fixed concentration of 100 μM (see Figure 3) and those that showed a kinase residual activity lower than 40% were tested also at a fixed concentration of 40 μM (see Figure 4).

IC_50_ values were calculated for compounds with a residual kinase activity lower than 40%. Compounds 37, 38, 52, 59,62 and 63 showed IC_50_ values in the micromolar range of 12.71 μM (9.57–16.80), 20.49 μM (17.46–24.08), 13.50 μM (12.47–14.62), 13.92 μM (11.89–16.29), 18.15 μM (16.78–19.64) and 24.86 μM (21.46–28.92), respectively. Remarkably, compound 28 shows a half-maximal inhibitory concentration of 3.31 μM (2.67–4.12). The IC_50_ curves for the sevenhits are reported on SI. The value of IC50 is based on the average of three independent measurements.

### 2.3. Molecular Recognition Studies of the Most Promising Fragment

To shed light on the possible recognition mechanism of the most effective inhibitor, compound 28 (IC_50_ = 3.31 µM) was investigated by mean of Supervised Molecular Dynamics simulations (SuMD). The primary scope was to assess if the hypothesized bound state obtained by our computational protocol was also accessible by simulating the fragment association from the unbound state without any information about the ligand conformation. Since in our VS-pipeline the pharmacophoric filter plays a primary goal in defining the bound geometries, its validation by using a more articulated technique based on MD and in which the water molecules need to be displaced by the fragment to reach the hinge region would provide the reliability of the binding mode. 

**Figure 5:**
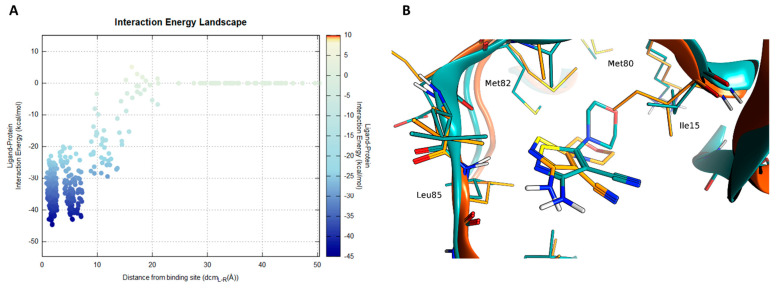
SuMD simulation of compound **28.** In panel (**A**) the interaction energy landscape is reported for the recognition trajectory displaying the ligand–protein interaction energy plotted against the distances between the protein–ligand center of mass. In panel (**B**), the superposition of the VS-pose (cyan) for compound **28** against the lowestenergy frame from the SuMD trajectory (orange).

A complete recognition pathway of the length of 15 ns is reported in VideoS2 (SI). Compound **28** showed three steps during the recognition, with two stable states (Figure 5A).

A pivotal role in the first phases (around 1 ns time mark) of the ligand recruitment within the binding site is played by Asp149, which acts as an electrostatic recruiter for the amino-thiophene moiety of the ligand. By contrast, the vicinal residue Lys38 hampers the ligand entrance into the core portion of the binding site due to the electrostatic repulsion between the charged amino group of the amino acid side chain and the non-charged amino group of the ligand. The balance in attraction and repulsion between the flexible side chains of these two amino acids located at the boundary of the binding site is depicted also by the large energetic funnel shown in Figure 5A at around 10 Å with regard to the distance between the centers of mass of the binding site and the ligand (dcm_L-R_).

Afterwards, the binding pathway is characterized by two stable ligand conformations within the binding site. The first state (S1) occurred at a dcm_L-R_ distance of 4.5 Å, with the ligand interacting with the backbone of Leu85 through its amino-thiophene moiety and the morpholine moiety oriented towards the external part of the binding site (solvent-exposed), while the second one (S2) at a dcm_L-R_ distance of 1.5 Å is characterized by a bivalent hydrogen bond with Leu85 and the morpholine moiety of the ligand buried within the hydrophobic selectivity pocket defined by Met80, Met82, Ile23 and the alkyl portion of the Lys38 side chain. Although these two states are characterized by similar interaction energy values (according to the AMBER forcefield), their energetic funnels have different shapes: the final state (S2) shows a narrower profile than the S1 state, suggesting that the pharmacophore binding mode (S2) has a higher stability than S1. Furthermore, the final bound state nicely retraced the pose obtained with the VS pipeline, validating both the pharmacophore model used in this work and the binding mode proposed by molecular docking for this compound (Figure 5B).

## 3. Discussion

**Figure 6:**
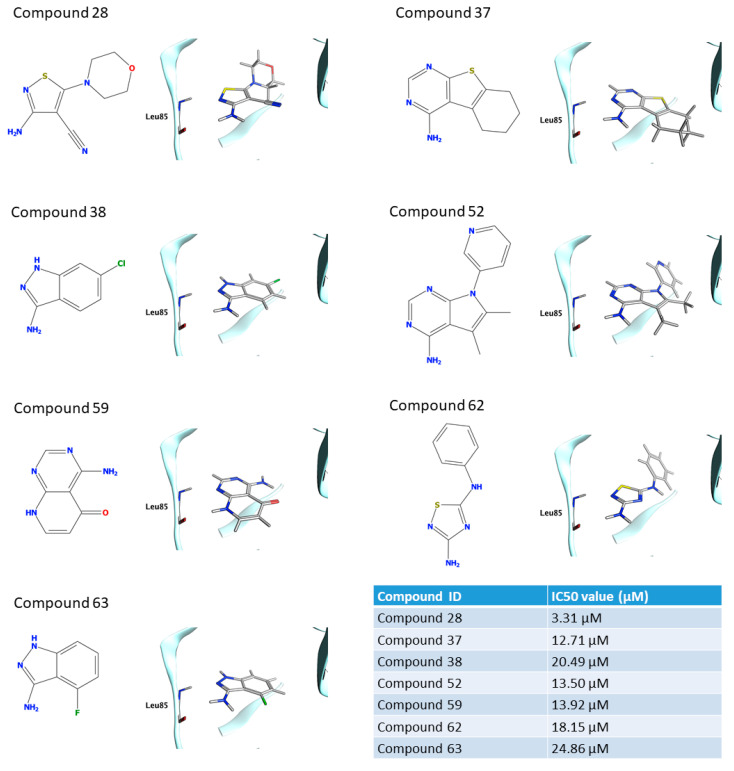
The structure and binding mode for the seven compounds for which the IC50 value is reported. The value of IC50 is based on the average of three independent measurements.

The seven fragments that were characterized by calculating the IC_50_ showed a noticeable chemical diversity including scaffolds spanning from one to three nitrogen-containing fused rings. The poses of the seven hits as obtained in the VS are reported in Figure 6. All the fragments logically share the common interaction pattern required by the pharmacophore filter. Interestingly, compounds 28, 37, 38, 52, 62, and 63 showed a similar interaction scheme in which an aromatic amine moiety was able to establish a hydrogen bond with the carbonyl oxygen of the Leu85 backbone while a further hydrogen bond between the Leu85 backbone amide is guaranteed by aromatic nitrogen in ortho to the amine group. Compounds **37**, **52**, and **59** share a conserved pyrimidine ring that is part of different fused systems. Compound **59** also has the pyrimidine ring in a different orientation: it restores the hydrogen bond donor by its fused pyridone ring. Compounds **38** and **63** present the same scaffold. To assess the novelty of the identified fragments, a substructure search was performed against ChEMBL using the main ring recognized by the pharmacophore as a query; except for compounds **38** e **52**, which resulted in **34** and **20** already known CK1δ inhibitors, for all the remaining hits none known inhibitors werefound sharing the principal ring. The 3-amino-indazole scaffold of compound **38** was found in a multikinase inhibitor (CHEMBL1999931) witha Kiof 316.23 nM [47]. For compound **52** a couple of ligands with low uM activity were found; in particular CHEMBL2000114 with a Ki of 1 uM arose from the same kinome scan from Abbott Labs [47]. Additionally, compound GSK1838705A showed the same scaffold of **52**, in this case the Ki reported is 3.5 uM but it is a residual activity since the compound is a potent inhibitor of ALK kinase (IC_50_ = 0.5 nM) [48].

## 4. Materials and Methods

### 4.1. Molecular Modelling and Docking

The virtual library used in this work was obtained through the merging of different libraries of commercially available compounds designed for FBDD. The vendors are Asinex (www.asinex.com, accessed on 25 July 2021) (Winston-Salem, NC, USA), Chembridge (www.chembridge.com, accessed on 25 July 2021) (San Diego, CA, USA), Enamine (www.enamine.net, accessed on 25 July 2021) (Kiev, Ukraine), Life Chemicals (www.lifechemicals.com, accessed on 25 July 2021) (Burlington, ON, Canada), Maybridge (www.thermofisher.com/it/en/home/chemicals/maybridge, accessed on 25 July 2021) (Worthing, UK), Otava (www.otavachemicals.com, accessed on 25 July 2021) (Ann Arbor, MI, USA), Timtec (www.timtec.net, accessed on 25 July 2021) (Newark, DE, USA), Vitas (www.vitasmlab.biz, accessed on 25 July 2021) (Miami, Fl, USA). The total number of fragments in the merged library is about 272,000 virtual compounds.

The merged library was prepared to be suitable for the Docking-Based Virtual Screening. This preparation consists of the following steps: the tautomeric state enumeration for each compound and determination of the most probable tautomer (for each molecule the three most tautomeric states was retained), the most probable ionization state at pH 7.4 calculation, the atomic partial charge calculation (using MMFF94 force field), the 3D coordinates generation. All these steps were performed using QUACPAC of theOpeneye suite [49] except for the 3D coordinated generation for which Corina Classic was used [50].

The protein used both for Docking and for MD simulation was prepared using MOE. The preparation consists ofthe removal of the crystallographic water molecules and other solvent molecules together with ions and the ligand. The correct protonation state for each residue at pH 7.4 was calculated with the Protonate3D tool of MOE.

For the Consensus Docking strategy, three different Molecular Docking protocols were used. To make the results more robust, the three docking protocols chosen rely on search algorithms of different types. The Molecular Docking Protocols are PLANTS [51,52,53] which is based on an Ant Colony Optimization algorithm, GOLD [54,55] which employs a genetic algorithm, and Glide [56,57] which use a systematic searching approach. The Scoring Functions adopted are CHEMPLP for PLANTS, ChemScore for GOLD, and Glide SP for Glide. For each fragment 50 poses were generated using each Docking Protocol even if the termination criteria and the nature of the algorithms did not always provide 50 poses, in particular for Glide SP.

Similarity and substructure searches were performed with MOE using the ChEMBL29 database.

### 4.2. Pharmacophore Modeling

Each ensemble of poses (one for each docking protocol) wasthen filtered using a pharmacophore model. This pharmacophore model was calculated using MOE: all the holo crystal structures available on the PDB for human CK1δ were superposed and the common features of each ligand were analyzed. The list of complexes included 23 complexes with PDB ID: 3UYT, 3UZP, 4HGT, 4HNF, 4KB8, 4KBA, 4KBC, 4KBK, 4TN6, 4TW9, 4TWC, 5IH5, 5IH6, 5MQV, 5OKT, 5W4W, 6F1W, 6F26, 6GZM, 6HMP, 6HMR, 6RCG, 6RCH. 

Since the ligands present in the crystal structures are drug-like molecules, it is difficult that a fragment can comply with all the common features observed in the crystal structures. For this reason (and because as stated above the first fragment identified in an FBDD process of a kinase inhibitor is a hinge binding fragment) the pharmacophore model was built using only the features involved in the interaction with the hinge region of the kinase. The model included three features: one hydrogen bond donor and one hydrogen bond acceptor to guarantee the interaction with the backbone of Leu85 (Figure 2). The last feature represents an aromatic ring also in the proximity of the hinge region. Only the molecule that has passed the Pharmacophore filtering for each protocol hwas retained (*consensus*).

### 4.3. Molecular Dynamics

The molecules retained after the consensus filtering were subjected to a post-docking refinement. The docking pose used in this step is the one obtained from Glide. All the simulations were carried out using ACEMD3 [58] with ff14SB as force field [59], the system preparation wasconductedwith MOE concerning protein preparation and with the use of AmberTools14 for the simulation box preparation.

For each complex, a simulation box was prepared: the protein was immersed in an explicit TIP3P [60] solvent box, with an ionic strength of 0.154M obtained using Na^+^/Cl^−^. The protein is 15Å away from the border of the box. 

Using the conjugate gradient method, the system energy was minimized for 500 steps; after this minimization the system was equilibrated in two stages. The first equilibration consists of 1ns of NVT simulation with harmonic positional constraints of 1 kcal mol^−1^Å^−2^ on the protein. In the second equilibration step, which consists in this case of 1ns of NPT simulation, the constraints of 1 kcal mol^−1^Å^−2^ were applied only on the α carbons of the protein. After the equilibration for each protein–pose complex, three NVT trajectories of 10 ns were produced. The average RMSF of the ligand during these three replicas was calculated and if this value wasgreater than 2Å the molecule was discarded.

A Supervised Molecular Dynamics [41,61] simulation was performed to gainsome insights into the binding process of the most potent fragment (Compound **28**). SuMD is an MD-based method developed to investigate molecular binding events without energetic biases. The algorithm is based on the supervision of the ligand–protein binding site center of mass distance during a classical short MD simulation. At the end of each small simulation (SuMD step), this distance is measured: if it hasshortened during the SuMD step, the simulation continues with another SuMD step, otherwise, it is stopped, and the simulation restarts from the previous set of coordinates. The fragment was placed 30 Å away from the protein. Each SuMD step was set to 300 ps.

### 4.4. Enzymatic Assay 

Compounds were evaluated towards CK1δ (aa 1-294, Merck Millipore, Frankfurter Strasse 250, Darmstadt, 64293, Germany) with the KinaseGlo^®^ luminescence assay (Promega Corporation, 2800 Woods Hollow Road Madison, WI 53711, USA) following procedures reported in the literature [22]. In detail, luminescent assays were performed in white 96-well plates, using the following buffer: 50 mM HEPES (pH 7.5),1 mM EDTA, 1 mM EGTA, and 15 mM MgCl_2_. Compound PF-670462 (IC50 = 14 nM) was used as a positive control for CK1δ [62] and DMSO/buffer solution was used as a negative control. In a typical assay, 10 μL of inhibitor solution (dissolved in DMSO at 10 mM concentration and diluted in assay buffer to the desired concentration) and 10 μL (16 ng) of enzyme solution were added to each well, followed by 20 μL of assay buffer containing 0.1% casein substrate and 4 μM ATP. The final DMSO concentration in the reaction mixture did not exceed 1%. 

After 60 min of incubation at 30 °C, the enzymatic reactions were stopped with 40 μL of KinaseGlo^®^ reagent (Promega). The luminescence signal (relative light unit, RLU) was recorded after 10 min at 25 °C using Tecan Infinite M100. Fixed-dose experiments were performed at 100 μM and for more potent compounds also at 40 μM. Two independent experiments were performed in duplicate and the corresponding residual activity of CK1δ was obtained. Data were analyzed using Excel and reported as the mean of the two experiments with standard deviation. For IC_50_ determination ten different inhibitor concentrations ranging from 100 to0.026 μM were used and each point was assessedin duplicate. IC_50_ values are the mean of three independent experiments and 95% confidence limits were also reported. Data were analyzed using GraphPad Prism software (version 8.0).

## 5. Conclusions

In the present work to find new potential CK1δ inhibitors, we elaborated a computational workflow for the identification of candidate hinge binding fragments. This workflow consists of the generation of a large number of poses for each compound of a virtual library of commercially available fragments using three different Docking protocols. These poses were filtered using a pharmacophore model and only the fragment for which each docking protocol was able to produce a pose that fits the model was retained (consensus docking). In the next, step each protein-fragment complex that passed the previous filter wassubjected to an MD-driven post-docking refinement to inspect the geometric stability of the pose. Finally, some fragments were manually selected among the group that demonstrated a good performance in the post-docking refinement; to validate the method these fragments were tested using an enzymatic assay test to assess the CK1δ residual activity, and for the most promising candidates, the IC_50_ value was determined, with a value in the low micromolar range. Five of seven fragments showed novel scaffolds for CK1δ, confirming that the proposed pipeline could be particularly useful to identify novel structures. 

## Supplementary Materials

The following are available online at https://www.mdpi.com/article/10.3390/ijms22189741/s1.
